# Transportable system enabling multiple irradiation studies under simultaneous hypoxia in vitro

**DOI:** 10.1186/s13014-018-1169-9

**Published:** 2018-11-13

**Authors:** Olli Metsälä, Joose Kreutzer, Heidi Högel, Petra Miikkulainen, Pasi Kallio, Panu M. Jaakkola

**Affiliations:** 1 Turku Centre for Biotechnology, University of Turku and Åbo Akademi University, Tykistökatu 6, FIN-20520 Turku, Finland; 2 Faculty of Medicine, Institute of Biomedicine, University of Turku, Kiinamyllynkatu 10, FIN-20520 Turku, Finland; 3 BioMediTech, Institute and Faculty of Biosciences and Engineering, Tampere University of Technology, Korkeakoulunkatu 3, FIN-33720 Tampere, Finland; 4 Turku PET Centre, University of Turku and Turku University Hospital, Kiinamyllynkatu 4-8, FIN-20521 Turku, Finland; 5 Helsinki University Hospital Comprehensive Cancer Center and Department of Oncology, University of Helsinki, Haartmaninkatu 4, FIN-00029 HUS Helsinki, Finland

**Keywords:** Hypoxia, Cancer, Radiotherapy, Radiation, In vitro, Minihypoxy

## Abstract

**Background:**

Cells in solid tumours are variably hypoxic and hence resistant to radiotherapy - the essential role of oxygen in the efficiency of irradiation has been acknowledged for decades. However, the currently available methods for performing hypoxic experiments in vitro have several limitations, such as a limited amount of parallel experiments, incapability of keeping stable growth conditions and dependence on CO_2_ incubator or a hypoxia workstation. The purpose of this study was to evaluate the usability of a novel portable system (Minihypoxy) in performing in vitro irradiation studies under hypoxia, and present supporting biological data.

**Materials and methods:**

This study was conducted on cancer cell cultures in vitro. The cells were cultured in normoxic (~ 21% O_2_) or in hypoxic (1% O_2_) conditions either in conventional hypoxia workstation or in the Minihypoxy system and irradiated at dose rate 1.28 Gy/min ± 2.9%. The control samples were sham irradiated. To study the effects of hypoxia and irradiation on cell viability and DNA damage, western blotting, immunostainings and clonogenic assay were used. The oxygen level, pH, evaporation rate and osmolarity of the culturing media on cell cultures in different conditions were followed.

**Results:**

The oxygen concentration in interest (5, 1 or 0% O_2_) was maintained inside the individual culturing chambers of the Minihypoxy system also during the irradiation. The radiosensitivity of the cells cultured in Minihypoxy chambers was declined measured as lower phosphorylation rate of H2A.X and increased clonogenic capacity compared to controls (OER~ 3).

**Conclusions:**

The Minihypoxy system allows continuous control of hypoxic environment in multiple wells and is transportable. Furthermore, the system maintains the low oxygen environment inside the individual culturing chambers during the transportation and irradiation in experiments which are typically conducted in separate facilities.

**Electronic supplementary material:**

The online version of this article (10.1186/s13014-018-1169-9) contains supplementary material, which is available to authorized users.

## Background

The lack of sufficient oxygen in tissues, i.e., hypoxia plays a central role in many physiological and pathological conditions. It is a major microenvironmental factor affecting cellular signalling in many diseases including cancer, stroke, atherosclerosis, inflammatory diseases such as rheumatoid arthritis, and neurodegenerative diseases [1, 2]. Several studies have shown that most solid tumours suffer from insufficient oxygen supply both acutely and chronically (reviewed in [3]). This is mainly caused by structurally and functionally abnormal vascularization of the tumour and limited diffusion capacity of oxygen. Hypoxia causes genome and proteome level changes in cancer cells leading to alterations in cell proliferation, cell cycle progression, metabolism, and cell survival and death (reviewed in [4]). Some of the changes restrict the tumour growth but hypoxia causes also a strong selective pressure towards a more aggressive tumour type.

Besides cancer progression, hypoxia also affects the outcome of cancer treatments including chemotherapy and radiotherapy [5]. The effect of radiotherapy relies partially on direct DNA damage but mainly on the oxygen radicals (ROS) formed by irradiation that requires molecular oxygen (reviewed in [6, 7]). Additionally, hypoxia also changes the positioning of the cells in cell cycle, which is a major factor affecting the responsiveness of the cancer cells to treatment including radiotherapy [8–10]. Many of the hypoxia responsive events are mediated by hypoxia-inducible factors (HIFs). HIFs are transcription factors governing the expression of over two hundred known genes involved in cell survival and metabolism, cell cycle progression and proliferation, motility and apoptosis (reviewed in [11]).

The conditions where in vitro studies are conducted would ideally correspond to the conditions in vivo as authentically as possible. Chemicals partially mimicking hypoxia such as cobalt chloride (CoCl_2_) as well as desferrioxamine (DFO) and dimethyloxalylglycine (DMOG) that act as inhibitors of prolyl 4-hydroxylases regulating the activity of HIFs but fail to mimic the tumour microenvironment. Various set-ups for achieving true hypoxic conditions in vitro have been established [12, 13]. The conventional method to induce hypoxia is to incubate cells in a tri-gas incubator or a hypoxia workstation. However, this restricts the types of treatments that can be performed in a low oxygen environment. Using workstations, the effect of hypoxia on radiosensitivity can only be studied by preincubating the cells in hypoxia to induce the adaptive system to low oxygen [14, 15]. The drawback of this method is that the cells are reoxygenated prior to irradiation. Therefore, it does not represent hypoxic tumour microenvironment. In addition, reoxygenation itself induces DNA damage [16], and thus interferes the detection of DNA damage caused by reoxygenation observed in tumours after radiotherapy [17–19].

To achieve mobility in the experiments and to expose cells to simultaneous hypoxia and irradiation, the most common solution is to use commercially available hypoxia containers. Here the cells are placed in a sealed container that is purged with a desired gas mix [20–25]. Similarly, sealed containers can be utilized to create anaerobic conditions or low oxygen conditions with oxygen scavengers [26, 27]. However, there is no independent temperature or further O_2_ or CO_2_ control. Therefore, the entire container with the cells inside needs to be incubated inside a normal incubator, and during the transportation and treatment, the temperature drops rapidly. In addition to commercially available products, there are also other solutions including a hypoxic atmosphere generated in a plastic bag [28] or in a self-made hypoxia containers [29]. Although budget-friendly, the oxygen conditions inside these types of hypoxia containers is difficult to maintain without feedback control [30]. Furthermore, the final oxygen level in the culture medium depends on the materials placed inside the chamber [21, 31]. Additionally, the hypoxia containers require high volume of expensive ready-made gas mixtures. Most importantly, large hypoxia containers exclude the use of multiple parallel experiments.

Here we present a novel system for performing hypoxia and especially hypoxic in vitro irradiation experiments. The portable Minihypoxy system consists of 1–6 individual 1-well culture chambers. Each chamber is independent from others and equipped with its own gas supply port. This enables flexible experiment planning where several different radiation doses and time points can be used within the same experiment. In our experiments, we have used a gas mixture supplied with 1% O_2_ but the partial oxygen pressure is convertible down to anoxic (< 0.1% O_2_) conditions. Our results show that the Minihypoxy system is functional and easy to use. The individual chambers can be disengaged from the system at least for 10 min without losing the hypoxic conditions or the desired temperature inside the chamber. This enables hypoxic in vitro irradiation experiments with multiple radiation doses and time points within one single experiment.

## Materials and methods

### Minihypoxy system

**Fig. 1:**
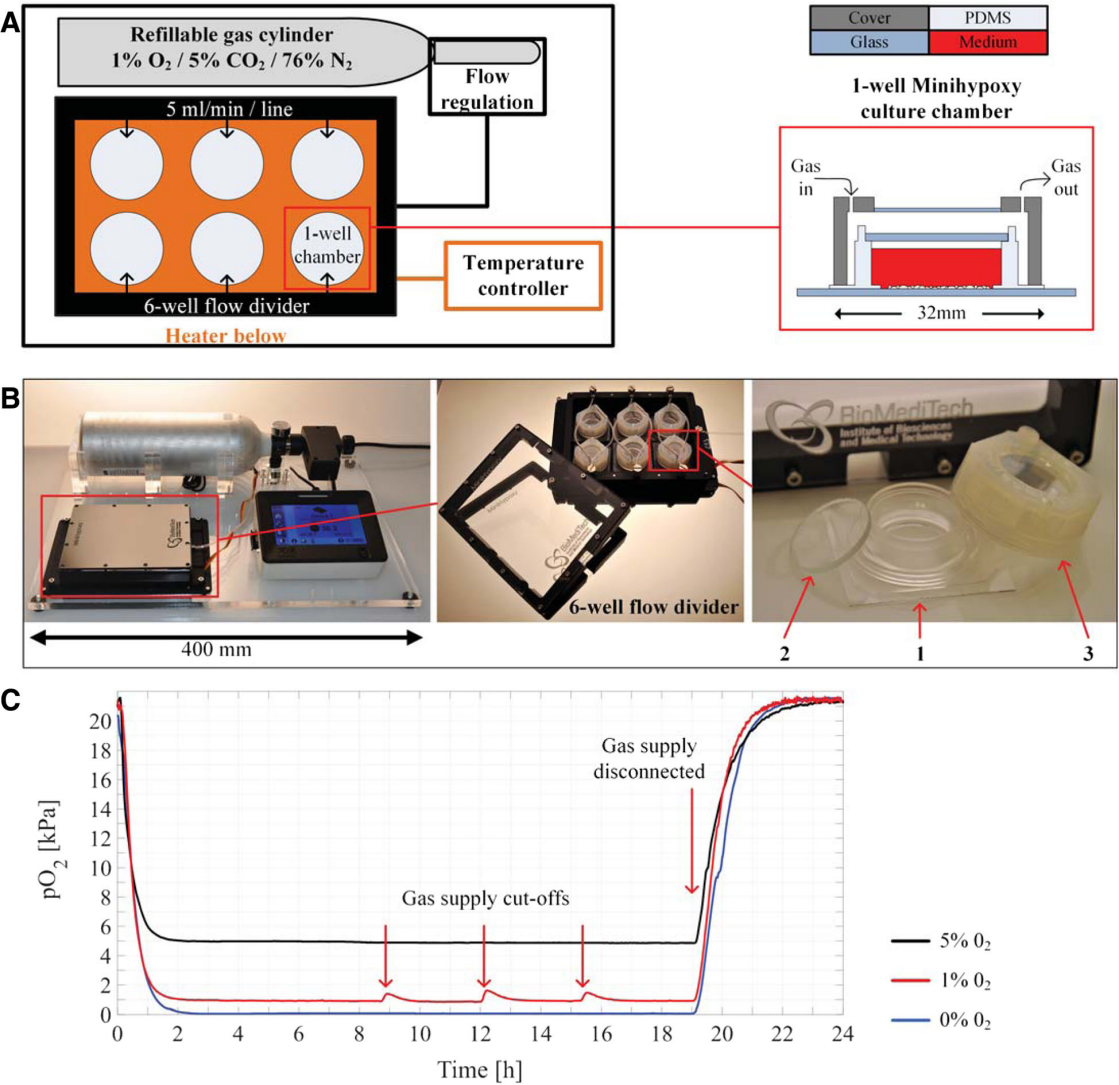
Structure and functionality of the Minihypoxy system. **a** A schematic diagram of the detailed structure of the system. The system consists of a refillable gas cylinder, a 6-well flow divider, a heater and a temperature controller, and six 1-well culture chambers (Minihypoxy chambers) made from polydimethylsiloxane (PDMS). **b** A picture of the Minihypoxy system, the 6-well flow divider and the Minihypoxy culture chamber on a glass substrate (1) with a lid (2) and a cover (3). **c** Dynamic response in the Minihypoxy culture chamber to descending and ascending oxygen level. The responses were tested on three different oxygen concentrations: 5% O_2_ (black line), 1% O_2_ (red line) and 0% O_2_ (blue line). Ascending oxygen level was reached by disconnecting the gas supply. The 1% O_2_ curve shows also the response to three short gas supply cut-offs

The Minihypoxy system is based on our previous studies [32, 33]. Briefly, it is a portable platform that maintains the desired atmosphere while transporting cells and, for example, during microscopy or irradiation experiments. The Minihypoxy system consists of a refillable gas cylinder (Catalina Cylinders, CA, USA), an ITO heater plate and a controller (Okolab s.r.l., Italy), a custom-made 6-well flow divider and 1-well culture chambers (Minihypoxy chambers) with covers and lids (Fig. 1a and b). The refillable gas cylinder can be loaded with any desired gas mixture. In this study, we used a gas mixture of 1% O_2_, 5% CO_2_, and 94% N_2_ (Linde gas, Riihimäki, Finland). Gas flow is regulated by adjusting the outlet pressure from the cylinder. In this study, we used a flow rate of 5 ml/min for all six supply lines (30 ml/min in total for six-wells). The operation time of the Minihypoxy systems with one fully loaded gas container for six Minihypoxy chambers is about 30 h. The gas mixture flows through the custom-made aluminium 6-well flow divider (SaloTeam Oy, Salo, Finland) to the Minihypoxy chambers. The ITO heater maintains the temperature in the desired temperature (37 °C in this study). The Minihypoxy culture chambers were fabricated as described in [32] using gas permeable polydimethylsiloxane (PDMS, Sylgard 184, Dow Corning, USA). Each chamber was sealed water-tightly using a glass lid (1 mm thick and 20.5 mm in diameter) from the top. The lid prevented evaporation and contamination inside the chamber. The cover is made of polymer by 3D printing (Keijom Oy, Pirkkala, Finland). The purpose of the cover is to maintain the desired gas environment around the PDMS culture chamber. Because of high gas permeability of PDMS, the gas mixture diffuses through the Minihypoxy chamber to the cells. The weight of the Minihypoxy system is 3.3 kg.

### Measuring oxygen concentration inside the Minihypoxy chamber

The oxygen concentrations inside the culture chamber were measured utilizing an in-house developed optical oxygen measurement setup [34]. Briefly, the Minihypoxy culture chamber was placed on top of a 0.5 mm thick glass substrate which contained oxygen sensitive fluorescent dyes. The chamber was filled with deionized water (1 ml), sealed using the lid and the cover, and placed on top of the ITO heater that maintained temperature at 37 °C. Optical reading of the fluorescent dyes in the Minihypoxy chamber was performed, through the ITO and glass substrates. The oxygen concentrations were measured using gas mixtures containing 0, 1 and 5% oxygen supplied from the prefilled gas cylinders. We also determined the dynamics of the oxygen concentration change in the Minihypoxy system when the gas supply was disconnected for a 5-min time period to mimic the irradiation treatment. All experiments were done with the flow rate of 5 ml/min.

### pH, evaporation and osmolarity of cell culture medium

The pH was measured as an end-point measurement utilizing a microFET pH meter (SI600with microFET sensor, Sentron, The Netherlands). Evaporation was estimated by determining the mass change during the experiment by weighting the wells before and after the experiment. OsmoMat 030 osmometer (GonoTec GmbH, Germany) was used for measuring the osmolarity.

### Cell culture

HeLa cervical cancer cell line and LNCaP prostate cancer cell line were obtained from ATCC (Rockville, MD, USA). UT-SCC74 primary cell line derived from human head and neck squamous cell carcinoma (HNSCC) was kindly provided by Prof Reidar Grénman. HeLa cells were cultured in Dulbecco’s Modified Eagle Medium (DMEM, Sigma-Aldrich), UT-SCC74 cells in Minimun Essential Medium Eagle with alpha modification (α-MEM, Gibco) and LNCaP cells in Roswell Park Memorial Institute medium (RPMI-1640, Gibco). All media were supplied with 10% fetal calf serum (FCS), L-glutamine and antibiotics (penicillin and streptomycin). Cells were cultured in 37 °C in air/5% CO_2_ and tested negative for mycoplasma once a month. The reference cells for the Minihypoxy system were cultured in 1% oxygen and 5% CO_2_ in the hypoxia workstation (Invivo2 400, Ruskinn Technology). The reference cells were plated on Minihypoxy culture chambers attached to glass substrate. Chambers were covered with the glass lid and kept on Petri dishes during incubation and for convenience in handling. For live cell imaging the cells were transfected with EGFP expression vector. Transfection reagent Lipofectamine 3000 (Invitrogen) was used according to manufacturer’s specifications. The images were obtained with LSM780 microscope (Carl Zeiss, Oberkochen, Germany).

### Protein detection and antibodies

For protein detection, western blotting and immunofluorescence staining were used. For protein expression analysis, cells were harvested in SDS-Triton lysis buffer. Protein concentration was measured using Bio-Rad DC Protein assay and protein detection using Pierce ECL Western blotting substrate (Thermo Scientific). Antibodies used were: HIF-1α (610959, BD Transduction Laboratories), phospho-H2A.X (Ser139) (2577, Cell Signaling), H2A.X (2595, Cell Signaling), GAPDH (5G4-6C5, HyTest), and β-actin (Ac-74, Sigma-Aldrich).

For immunofluorescence staining, cells were fixed with ice cold methanol. The DNA double strand breaks were visualized using antibody against phosphorylated H2A.X (γH2A.X) (2577, Cell Signaling). The nuclei were stained using Hoechst 33342 (Invitrogen). Cells were imaged with Olympus BX60 (40X). The intensity of the phosphorylated H2A.X staining per cell (minimum 4 optical fields yielding minimum 150 cells per condition) was measured using ImageJ software (NIH, USA). Experiments were done as parallel treatments and each experiment was repeated at least three times.

### Irradiation

Irradiations were performed using the Faxitron Multirad 350 (Faxitron Bioptics, LLC) located in Turku PET Centre at the Turku University Hospital and University of Turku, Finland. The voltage was set to 250 kV with a current of 14 mA (dose rate 1.28 Gy/min ± 2.9% at 50 cm distance from the radiation source). The X-ray beam was hardened with a 0.3 mm copper filter. Cells were exposed to 2 or 6 Gy doses. The control samples (0 Gy) were sham irradiated.

The cells were transported to the irradiator in protected boxes (normoxic and hypoxic preconditioned references) or in the Minihypoxy system. This minimized the possible stress caused by temperature variations. Radiation exposures were conducted at room temperature. The Minihypoxy chambers were disconnected from the flow divider and placed in the irradiator. After the irradiation, the Minihypoxy chambers were connected back to the Minihypoxy system. Reference cultures were placed into a standard incubator for 1 h before the sample collection.

### Clonogenic assay

To study the radiosensitivity of the cells, clonogenicity was determined. First, to find an optimal cell amount for the assay, the plating efficiency (PE) was determined. Briefly, cells were plated at different densities on 6-well plates (100–1000 cells/ml) and cultured for seven days at standard cell culturing conditions (+ 37 °C in air/5% CO_2_). The colonies were stained using crystal violet and colonies (> 50 cells) were counted using Image J. The PE was calculated by dividing the average colony count with cells plated (PE = no of colonies formed/no of cells seeded * 100). For the actual radiosensitivity experiment, density of 750 cells per well was selected.

The cells were seeded into the Minihypoxy chambers and incubated in 1% O_2_ (Minihypoxy and preconditioned reference in the hypoxia workstation) or 21% oxygen (normoxic reference in a standard incubator) for 24 h. The cells were irradiated at doses of 0, 2 or 6 Gy and immediately re-plated on a 6 well plate at 375 cells/ml, 2 ml per well for clonogenic assay and cultured in standard cell culturing conditions for seven days. Staining and quantification was performed as described above. The survival fraction was calculated using formula SF = no of colonies formed after treatment/(no of cells seeded * PE) as described previously [35].

## Results

### Partial oxygen pressure (pO_2_) inside the Minihypoxy culture chamber

The Minihypoxy system consists of a refillable gas cylinder, a heat plate and a controller, a custom-made 6-well flow divider and 1-well culture chambers (Minihypoxy chambers) (Fig. 1a and b). A gas mixture flows through the 6-well flow divider to the Minihypoxy chambers. The heat plate maintains the temperature in the desired temperature (37 °C in this study). The Minihypoxy culture chambers were sealed using glass lids and the whole chamber with the lid is covered by a polymer-made cover. A more detailed description of the system is provided in materials and methods.

To demonstrate the dynamic response of the Minihypoxy system to changes in oxygen supply, we followed the oxygen concentration at the bottom of the Minihypoxy culture chamber during the descending and ascending oxygen step response and during the gas supply cut-offs. As shown in Fig. 1c, the oxygen concentration reached the set point relatively fast. The time constants for the descending oxygen step responses from ambient oxygen level to the target concentration values of 5, 1 and 0% were 21 min, 21 min and 29 min, respectively. The oxygen concentration remained at the same level during the experiment and was maintained as long as the gas supply was connected. During the 5 min of gas supply cut-off, the oxygen concentration rose approximately 0.6 percentage points but returned to the set point after 1 h. When the Minihypoxy culture chamber was entirely disconnected from the gas supply, the time constants of the ascending oxygen concentration from 5, 1 and 0% to the ambient oxygen level was 52 min, 46 min and 56 min respectively.

### pH, evaporation and osmolarity measurements

To show that the Minihypoxy culture chamber sealed with a lid and a cover maintains desired cell culturing conditions also during the irradiation treatment, we plated HeLa cells in the Minihypoxy culture chambers and incubated them either in the Minihypoxy system, in a hypoxia workstation (Invivo2, Ruskinn Technology), or a standard CO_2_ incubator. The chambers were irradiated, and culture media was collected to measure the pH, evaporation and osmolarity of the culturing media after irradiation.

The measurements show that the evaporation was slightly smaller in the Minihypoxy system than inside the hypoxia workstation or in the incubator (Table 1). No change was observed in the osmolarity between the different culturing conditions. The pH increased at a relatively high level in cultures incubated in the hypoxia workstation or in the incubator, while the pH measured from media of the Minihypoxy system remained significantly lower (7.18 vs. 7.51 and 7.65). This is probably due to the continuous gas supply and the regulated constant temperature throughout incubation in the Minihypoxy system, while the chambers from hypoxia workstation and incubator are lacking a steady CO_2_ supply to buffer the media during irradiation.

**Table 1:** Evaporation, pH and osmolarity measured from the 1-well culture chambers on glass substrate in the Minihypoxy system, the hypoxia workstation (hypoxia WS) and the standard incubator (*n* = 6)

		Evaporation [ul]	Evaporation [ul/h]	pH	Osmolarity [mOsm]
Minihypoxy system (*n* = 6)	mean (std)	59 (14)	2.46 (0.57)	7.18 (0.06)	0.309 (0.004)
1-well in hypoxia WS (*n* = 6)	mean (std)	94 (46)	3.90 (1.91)	7.51 (0.09)	0.306 (0.002)
1-well in incubator (*n* = 6)	mean (std)	69 (40)	2.89 (1.67)	7.65 (0.02)	0.297 (0.002)
Fresh Medium (*n* = 6)	mean (std)	-	-	7.38 (0.01)	0.298 (0.001)

### Hypoxia is generated and maintained inside the Minihypoxy chamber for a prolonged time

**Fig. 2:**
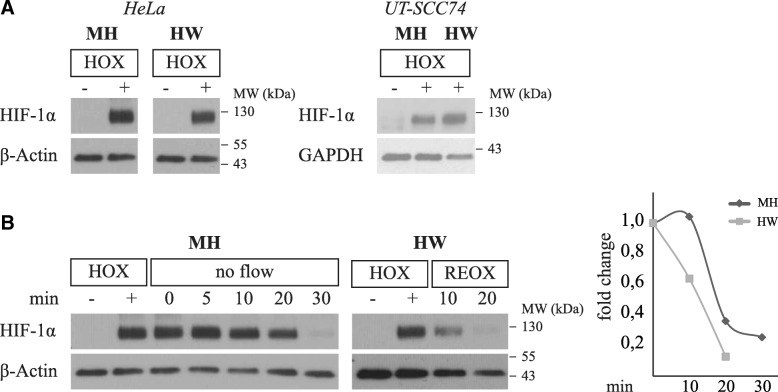
Hypoxic conditions are maintained in Minihypoxy chambers. **a** Hypoxia-inducible factor HIF-1α is equally induced in Minihypoxy system (MH) and in hypoxia workstation (HW) in 1% O_2_ (HOX). The hypoxic response is shown with two carcinoma cell lines (HeLa, UT-SCC74). **b** The Minihypoxy chambers (MH) were disconnected from the gas flow (no flow) and the level of HIF-1α was followed for 30 min. The control cells were cultured in hypoxia workstation (HW) for 24 h and then transferred to normal cell incubator with 21% O_2_ (REOX). The samples were collected accordingly

In addition to measuring the partial oxygen pressure (pO_2_) inside the Minihypoxy chamber, we determined biological effects on cells cultured in a Minihypoxy chamber by using hypoxia-inducible factor 1 alpha (HIF-1α) as a marker protein since it serves as a sensitive indicator for hypoxia. HIF-1α is induced by hypoxia and is rapidly degraded in response to increased oxygen supply (> 5% O_2_) [36–39]. To ensure the effective HIF-1α induction in the Minihypoxy chambers supplied with 1% O_2_, HeLa and UT-SCC74 cells were cultured in the Minihypoxy chamber for 24 h. Both cell lines showed a marked induction of HIF-1α level (Fig. 2a). The magnitude of the induction was in line with the level of HIF-1α determined from cells cultured in the hypoxia workstation in 1% O_2_ for 24 h.

In in vitro radiotherapy studies, the cells are disconnected from the Minihypoxy flow divider and placed into the irradiator for approximately 5 min depending on the radiation dose. To study how long the Minihypoxy chambers maintain the hypoxic microenvironment inside the chamber without the gas flow, we performed an experiment where the chambers were disconnected from the Minihypoxy flow divider. After 24-h culturing, samples were collected at four time points (5, 10, 20, and 30 min) after cut-off of the gas flow (Fig. 2b). The reference samples were cultured in hypoxia workstation (1% O_2_) for 24 h and then transported to a standard incubator with ~ 21% oxygen. The level of HIF-1α was significantly higher after 5, 10 and 20 min of removing gas flow in cells cultured in the Minihypoxy chamber as compared to reoxygenated reference samples (Fig. 2b). Together with the measurements showing the changes in the partial oxygen pressure inside the chamber (Fig. 1c), this indicates that the Minihypoxy chamber sealed with the lid and the cover maintains hypoxia up to 20 min without the gas flow.

### Enhancement of cellular radiation resistance under hypoxia in the Minihypoxy chambers

**Fig. 3:**
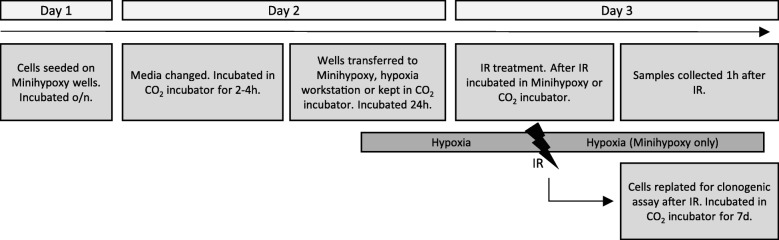
Work flow chart on in vitro irradiation experiments

**Fig. 4:**
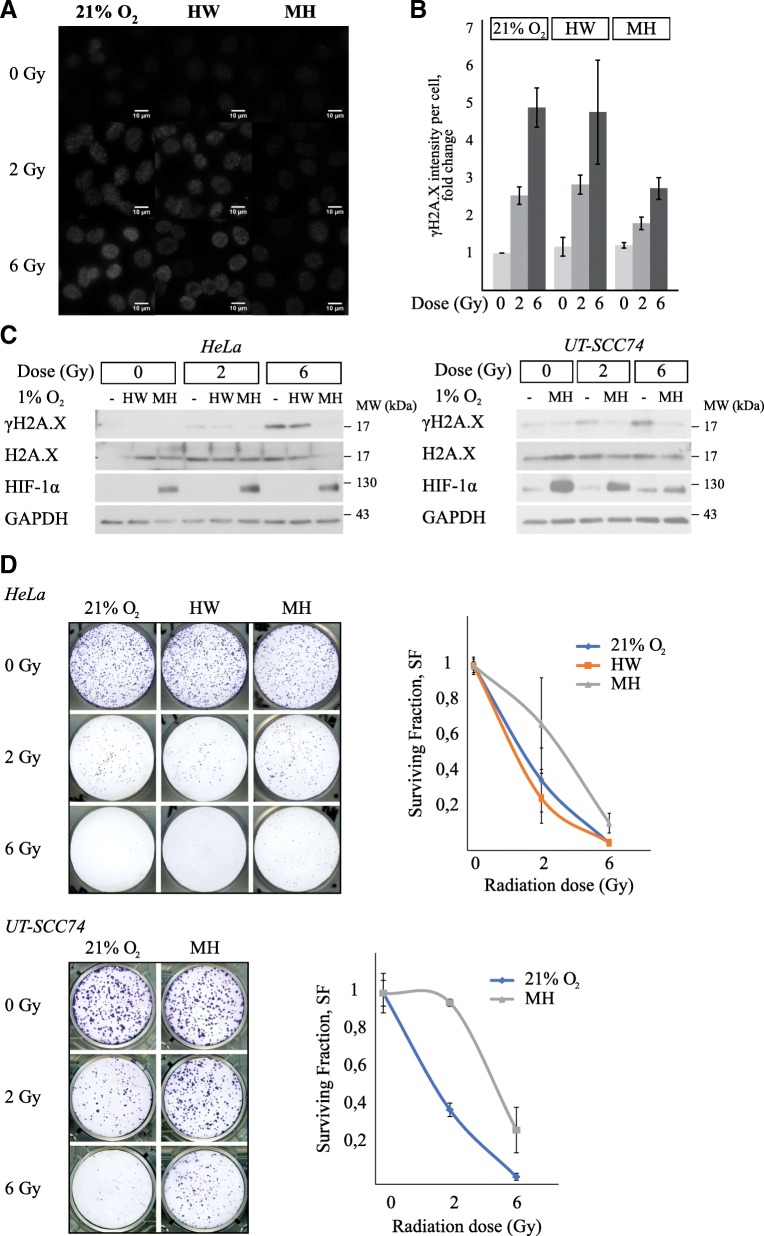
The radiosensitivity of cells treated in Minihypoxy chambers is decreased. **a** The phosphorylation of H2A.X histone (γH2A.X) marks the double strand breakages on. The γH2A.X level is gradually increased in HeLa cells with dosage in normoxia (21% O_2_) and in hypoxia preconditioned samples (HW) but in Minihypoxy samples (MH) there is significantly less damage. **b** Quantification of three independent biological experiments (*n* = 3). **c** The western blots showing γH2A.X level in HeLa and UT-SCC74 cells. Representative blot is shown. **d** The clonogenicity of the HeLa and UT-SCC74 cells is decreased in all conditions but especially in normoxic (21% O_2_) and in hypoxia preconditioned (HW) samples (HeLa only). Quantification of three independent biological experiments (*n* = 3)

To test the Minihypoxy system for simultaneous hypoxia and irradiation, we determined the sensitivity of cultured cells to radiotherapy in two oxygen conditions (1 and 21%). The experiments included determining the level of DNA damage and cell growth studies (work flow presented in Fig. 3). Several previous studies have demonstrated that hypoxia causes resistance to radiation and thus shows less DNA damage as measured by serine phosphorylation (Ser139) of H2A.X histone (γH2A.X), which is commonly used as an indicator of double strand DNA breakage [10, 40]. The cells were plated on the Minihypoxy chambers and cultured in 1% O_2_ for 24 h prior to irradiation. The reference samples were cultured in 21% O_2_ or in the hypoxia workstation in 1% O_2_ for 24 h (hypoxic preconditioning). The cells were irradiated at single doses of 0, 2 and 6 Gy, and samples for immunostaining and western blot analysis were collected one hour after irradiation (Fig. 3). As expected, immunostaining revealed a dose-dependent increase in the intensity of γH2A.X on HeLa cell nuclei 1 h after irradiation under normoxia and hypoxic preconditioning. The increase in γH2A.X level was irradiation dose-dependent and did not differ between normoxic and preconditioned samples (HW) (Fig. 4a and b). In striking contrast, the response to irradiation was strongly diminished in the Minihypoxy chamber under 1% O_2._ To reach the same treatment response in a hypoxic Minihypoxy chamber as in 21% O_2_ or preconditioned conditions, the estimated value of radiation dose would need to be approximately three times higher (oxygen enhancement ratio, OER~ 3). The estimation is based on the comparison of the γH2A.X levels in different conditions (Fig. 4b). A similar response was seen in western blot analysis of both HeLa and UT-SCC74 cells. A clear radiation dose-dependent increase in the level of phosphorylated H2A.X seen in normoxic and hypoxia preconditioned (HW) samples was strongly attenuated in hypoxic Minihypoxy chamber (Fig. 4c).

Finally, we determined the clonogenic capacity of the irradiated cells. The cells were irradiated as previously described and plated for clonogenic assay immediately after irradiation (Fig. 3). The clonogenic capacity of the cells decreased rapidly in response to irradiation (Fig. 4d). However, the cells irradiated in the Minihypoxy chambers retained some of their clonogenicity even after 6 Gy dosage, whereas the reference cells grown under normoxia (21% O_2_) or preconditioned in the hypoxia workstation (HW) lost their clonogenic potential completely (Fig. 4d). The effect was even more prominent in primary human head and neck carcinoma UT-SCC74 cells (Fig. 4d). Taken together, the data indicate that the Minihypoxy system can mimic the hypoxic microenvironment and maintains hypoxia for prolonged time also when transported and placed inside an irradiator.

## Discussion

Hypoxia is one of the major microenvironmental factors affecting carcinogenesis and cancer progression and is characteristic for many pathologies such as inflammation, inflammatory diseases as well as stem cell growth. Moreover, some normal tissues can be considered as permanently hypoxic as the partial oxygen pressure is constantly lower than 5%. Yet, most of the in vitro research on cell cultures is still conducted under partial oxygen pressure up to 21%. There is an obvious need for cost-effective and easy-to-use application for a device with adjustable oxygen concentration. Ideally, all laboratories especially those working on the research fields of cancer, stem cells and inflammation, should have access to the use of a hypoxia incubator.

Here we present a novel device for conducting in vitro experiments in hypoxia. Our results show that experiments in the Minihypoxy system are reproducible and reliable, and results are comparable to studies performed in a hypoxia workstation. Furthermore, the Minihypoxy system maintains hypoxia also when transported to another location as well as when used inside an irradiator. Our results show that performing radiation experiments in the Minihypoxy system markedly reduces the radiosensitivity of carcinoma cells determined as reduced DNA damage and attenuated loss of clonogenic capacity, thus presenting its utility in in vitro radiosensitivity measurements.

There are several advantages of the Minihypoxy system over conventional hypoxia workstations or hypoxia chambers. First, media in the Minihypoxy culture chamber reaches 1% oxygen concentration within 2 h, which is relatively fast as compared to media placed into a hypoxia workstation where it may take up to 24 h for media to reach the set point [41]. The Minihypoxy chambers are made of silicone elastomer which is gas permeable material and therefore enhances the gas exchange inside the chamber. Second, in the Minihypoxy system, the cells are steadily kept on a heat plate, which eliminates the stress effects caused by the temperature changes. Even small temperature changes might affect significantly the behaviour of cells [42, 43]. Third, the pH changes in the Minihypoxy system are minimized compared to other hypoxia systems as the constant CO_2_ supply maintains the buffering capacity of the media. Even inside a standard incubator, the conditions may vary if the incubator is heavily used. In addition, the closed structure of the Minihypoxy culture chamber decreases the evaporation and prevents the concentration of the culture media. Evaporation increases the osmolarity of the cell culture media and has a negative effect on cells [31, 44]. In other words, the optimal composition of the culture media can be maintained in the Minihypoxy system. Fourth, the Minihypoxy system also enables live cell imaging under low oxygen conditions as the chambers are designed with undisrupted optical path (Additional file 1: Figure S1). Everyday monitoring of the cells while in culture can be performed with the adjacent heat plate. Therefore, the cells can also be observed and imaged during the long-term hypoxia culturing. Moreover, high resolution live cell imaging of individual Minihypoxy chambers requires heated microscope chamber to keep the temperature stable during a long-term imaging experiment. Most importantly, the Minihypoxy system is transportable and allows experimental planning with several different independent irradiation treatments and time points at the same time.

The sample collection procedure is similar in hypoxia containers and Minihypoxy culture chambers as opening of the container or the chamber exposes the cells to a short period of reoxygenation stress. A benefit of the Minihypoxy chambers compared to the hypoxia containers is that the Minihypoxy chambers can be handled one chamber at a time while the conditions in the other chambers are not affected. We found no difference in the level of the marker protein HIF-1α between the samples collected using non-preconditioned washing buffer versus samples where preconditioned buffer was used or which were collected inside the hypoxia workstation (Additional file 2: Figure S2). However, as many experimental setups would benefit from a perfusion system, this need has been taken under consideration when developing the next generation Minihypoxy chambers. Taken together, the Minihypoxy system offers many benefits over current methodologies for creating low oxygen atmosphere for cell culture experiments in a compact device. It also provides benefits compared to other hypoxia solutions in regards its mobility and capability of maintaining optimal cell growth conditions during transportation.

## Conclusions

The Minihypoxy system provides a transportable easy-to-use platform for controlling oxygen concentration in in vitro cell cultures. In addition to the oxygen level, the Minihypoxy system also maintains the temperature and pH of the culture constant. Here we present the utility of the Minihypoxy system in in vitro irradiation experiments on carcinoma cell cultures under reduced oxygen level. Besides simultaneous hypoxia and irradiation the transportability of the system enables other experimental designs performed under hypoxia.

## Additional files

Additional file 1: **Figure S1.** Live cell images of EGFP expressing prostate cancer cell line LNCaP cells. The images were obtained using 10× and 63× magnifications. (PDF 1137 kb)

Additional file 2: **Figure S2.** The samples can be collected without performing hypoxic preconditioning on the washing buffer. The cells were washed twice before collecting using either PBS stored in normal 21% oxygen concentration (N) or preconditioned for 24 h in 1% oxygen concentration (pre). As a control there is a sample which is collected inside the hypoxia incubator using preconditioned washing buffer (H). (PDF 207 kb)

## Abbreviations

PE: Plating efficiency
ROS: Reactive oxygen species, oxygen radicals
SF: Surviving fraction
