# Supplementary material for: Transportable system enabling multiple irradiation studies under simultaneous hypoxia in vitro
Source: Radiat Oncol. 2018 Nov 13;13:220. doi: 10.1186/s13014-018-1169-9 (PMC6234660; doi:10.1186/s13014-018-1169-9)
Supplement: Supplementary file 1 — Figure S1. Live cell images of EGFP expressing prostate cancer cell line LNCaP cells. The images were obtained using 10× and 63× magnifications. (PDF 1137 kb) [file 13014_2018_1169_MOESM1_ESM.pdf]

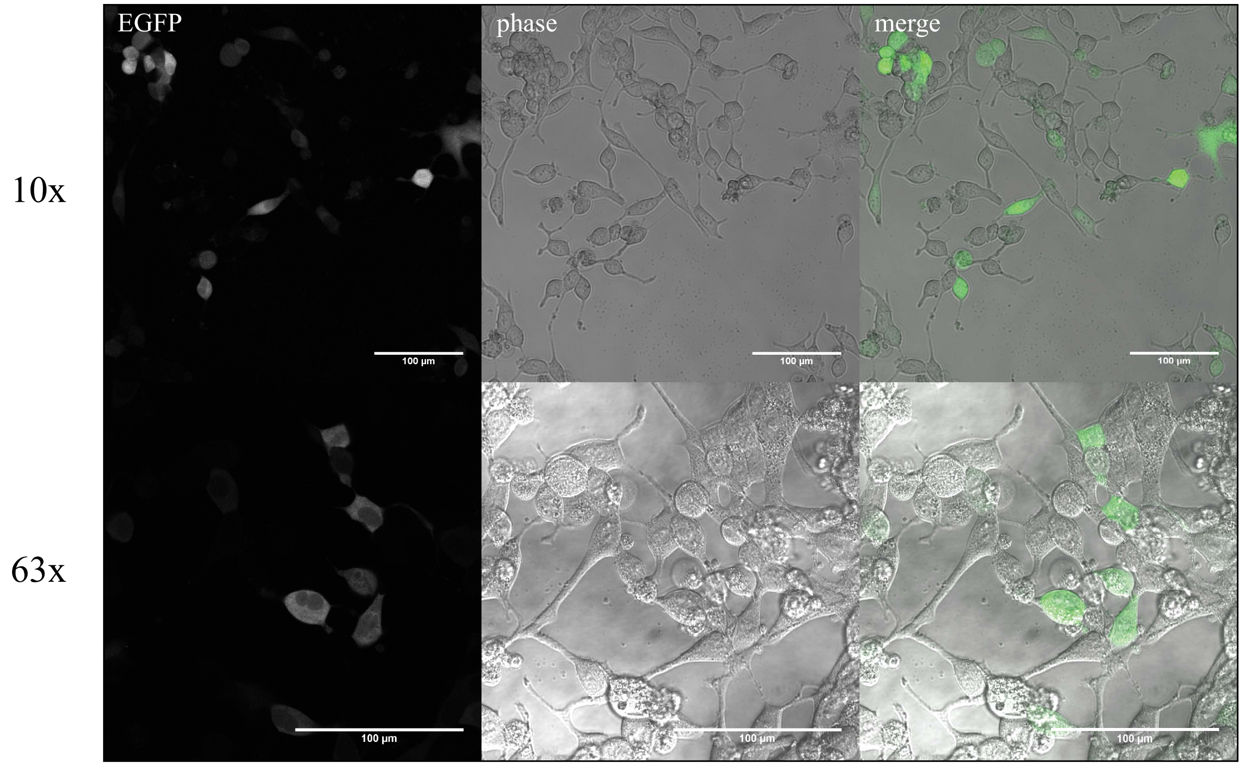

Figure S1: Live cell images of EGFP expressing prostate cancer cell line LNCaP cells. The images were obtained using 10x and 63x magnifications.
