# Supplementary material for: Transportable system enabling multiple irradiation studies under simultaneous hypoxia in vitro
Source: Radiat Oncol. 2018 Nov 13;13:220. doi: 10.1186/s13014-018-1169-9 (PMC6234660; doi:10.1186/s13014-018-1169-9)
Supplement: Supplementary file 2 — Figure S2. The samples can be collected without performing hypoxic preconditioning on the washing buffer. The cells were washed twice before collecting using either PBS stored in normal 21% oxygen concentration (N) or preconditioned for 24 h in 1% oxygen concentration (pre). As a control there is a sample which is collected inside the hypoxia incubator using preconditioned washing buffer (H). (PDF 207 kb) [file 13014_2018_1169_MOESM2_ESM.pdf]

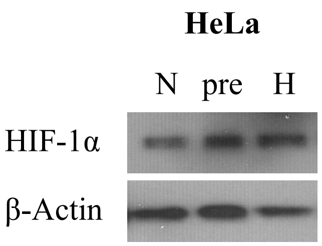

Figure S2: The samples can be collected without performing hypoxic preconditioning on the washing buffer. The cells were washed twice before collecting using either PBS stored in normal 21% oxygen pressure (N) or preconditioned for 24 hours in 1% oxygen pressure (pre). As a control there is a sample which is collected inside the hypoxia incubator using preconditioned washing buffer (H).
